# The cost-effectiveness analysis of JinQi Jiangtang tablets for the treatment on prediabetes: a randomized, double-blind, placebo-controlled, multicenter design

**DOI:** 10.1186/s13063-015-0990-9

**Published:** 2015-11-03

**Authors:** Xiao Sun, Liping Guo, Hongcai Shang, Ming Ren, Yue Wang, Da Huo, Xiang Lei, Hui Wang, Jingbo Zhai

**Affiliations:** Tianjin University of TCM, 9 ShuangfengStreet, Nankai District, Tianjin, 300193 China; Baokang Hospital of Tianjin University of TCM, Tianjin, China; Dingzhi Men Hospital of Beijing University of TCM, Tianjin, China; Inner Mongolia Autonomous Region People’s hospital, Tianjin, China; Tianjin University of TCM Affiliated Wuqing Hospital of TCM, Tianjin, China; Tianjin Institute of Clinical Evaluation, Tianjin University of TCM, 88 YuquanStreet, Nankai District, Tianjin, 300193 China

**Keywords:** Cost-effectiveness analysis, JinQi Jiangtang tablets, Prediabetes

## Abstract

**Background:**

At present, diabetes is a chronic disease of great cost and heavy burdens. The International Diabetes Federation has repeatedly warned that by 2025, the global number of diabetics would rise to 333 million from 194 million in 2003. Although the occurrence of diabetes in developing countries is lower, China has a large population, so that the number of cases is increased. At the same time, more people have prediabetes, a growing health concern where a large percentage of the patients develop full type 2 diabetes. In addition, the patients of diabetes easily incur complications such as blindness, kidney failure, and cardiovascular diseases that can seriously affect the patients’ quality of life and cause great economic burdens to family and society. Therefore, effective interventions for prediabetes are needed to prevent or delay the occurrence and development of diabetes.

**Methods:**

A randomized controlled trial that was assessed with pharmacoeconomic methods was undertaken in this study. The study term was 24 months (12 months for the intervention and 12 months for follow up). Four hundred participants, recruited from four cities in China: Beijing, Tianjin, Xian, and Naning, were randomized to the treatment group (JQJT tablets) and the control group (placebo). Participants included in this study had been diagnosed with prediabetes according to the criteria for western medicine and Traditional Chinese Medicine (TCM). The end-point effectiveness indexes included the incidence of diabetes and the reversion rate. The drug costs and lifestyle intervention costs were included in the total costs. The study used the cost-effectiveness analysis to discuss the economic advantage of the JQJT tablets.

**Results:**

The outcomes of the study contained 2 sections,namely clinical outcomes and cost-effectiveness analysis outcomes. The clinical outcomes: the treatment group and control group had no significant statistical difference P> 0.05) on the baseline of situation; Jinqi Jiangtang tablet effectively reduced the incidence of diabetes mellitus and enhanced reversion rate. compared with the control group (p< 0.05); the scores of SF-36 of two groups had no significant difference P> 0.05); finally the compliance of participants between the two groups had no signicant difference. The cost-effectiveness analysis outcomes:in the intervention period of 12 months,on the aspect of reversion rate, the treatment group had better economic advantage by using cost-effectiveness ratio and the incremental cost-effectiveness ratio;on the aspect of the incidence of diabetes, the control group had better economic advantage by using cost-effectiveness ratio and the incremental cost-effectiveness ratio; in the follow-up peiod of 24 months, on the aspect of reversion rate, the treatment group had better economic advantage by using cost-effectiveness ratio and the incremental cost-effectiveness ratio, on the aspect of the incidence of diabetes, the control group had better economic advantage by using cost-effectivenes ratio and the incremental cost-effectiveness ratio.At the same time, these outcomes remained the same by sensitivity analysis. Assuming that prices and resident incomes rose 5%, the sensitiveness analysis shows that the two group affected by the paremeters changed little.

**Conclusion:**

The importance and effectiveness of lifestyle education and JinQi Jiangtang tablets was proven. In both the intervention period and follow-up, JinQi Jiangtang tablets combined with lifestyle education had a greater cost advantage effect than the lifestyle education alone on the reversion rate; the lifestyle education had a greater cost advantage effect than the JinQi Jiangtang tablets combined with lifestyle education on the incidence of diabetes.

**Trial registration:**

Chinese Clinical Trials ChiCTR-TRC-09000401) , registered on 25 May 2009.

**Electronic supplementary material:**

The online version of this article (doi:10.1186/s13063-015-0990-9) contains supplementary material, which is available to authorized users.

## Background

At present, diabetes is a chronic disease that incurs great costs and heavy burdens. The International Diabetes Federation has repeatedly warned that by 2025, the global number of diabetes cases will have risen to 333 million from 194 million in 2003. In China, the number of diabetes cases will be 46 million, and the diabetes burden will be expected to 450.1 billion yuan. Some studies have discovered that about 5 % to 10 % of prediabetes will develop into diabetes within 1 year. Therefore, effective interventions for patients with prediabetes that can prevent and delay the occurrence or development of diabetes, thereby incurring an economic advantage, is necessary.

### Prevention and treatment on prediabetes in lifestyle and Traditional Chinese Medicine

The Daqing study from 1986 to 1994 discovered that lifestyle interventions could decrease the incidence of diabetes by 30 % to 50 % [1]. Nineteen randomized controlled trials, which included a systematic evaluation of prediabetes interventions, showed that the combination of lifestyle and Chinese medicine intervention was able to decrease fasting blood glucose in 1471 cases. Furthermore, the 2-hour postprandial insulin levels and 2-hour postprandial blood glucose levels were lower than observed with simple lifestyle intervention [2]. Therefore, a combination of lifestyle and Traditional Chinese Medicine (TCM) interventions offer possibilities for the effective prevention of prediabetes.

### Pharmacodynamics and preclinical use of JinQi Jiangtang tablets

Pharmacodynamics and preclinical use of JQJT tablets, produced by Coptis, Astragalusand Honeysuckle, suggested that the tablets could be beneficial for the treatment of prediabetes. Regarding aspects of pharmacodynamics, some studies have proven that the tablets can affect glycometabolism, insulin resistance (IR), and serum insulin, as well as immune function [3–5]. On the other hand, Shen Zhufang discovered that JQJT tablets can improve insulin resistance in humans [6]. Zhang Rongrong indicated that JQJT tablets can delay the development of diabetic nephropathy and prevent progression from prediabetes to diabetes [7].

## Methods

### Objective

The objective of this study was to evaluate the effectiveness,safety, and economic advantage of JQJT tablets for prediabetes in China by comparison with a placebo.

### Research type

This was a randomized, controlled, double-blind clinical trial. Ethical approval was given by the Chinese Ethics Committee for Registering Clinical Trials (ChiECRCT) with the following reference number: ChiECRCT-20090009. The trial was registered at the Chinese Clinical Trial Registry (Registration ID:ChiCTR-PRC-09000401), and the protocol, the informed consent form, and other relevant documents were reviewed and approved by its affiliated Ethics Review Committee. The Tianjin University of Traditional Chinese Medicine was held responsible for the organization and coordination of the trial sites and staff. The participating institutions included the First Hospital of Peking University, the Second Affiliated Hospital of Tianjin University of TCM, the Metabolic Diseases Hospital of Tianjin Medical University, the first Affiliated Hospital of Guangxi Medical University and Xijing Hospital.

### Randomized controlled trial

The randomization of the trial was completed by the independent data center Interact Voice Responding System (IVRS). According to a random sequence table (generated by SAS 8.2), patients who conformed to the inclusion criteria were allocated randomly into one of the two groups in a ratio of 1:1. The identification code and random number, which were unique for each patient, were generated by the IVRS. This study was designed to be double blind. After the database was closed, the person in charge of the blind code delivered an allocation schedule designated only as groups A and B to the statistics department. Then, after receiving the statistical outcome and final report, the person in charge of the blind code declared the real meaning of groups A and B.

### Ethics

The study was conducted in abidance with Chinese clinical trial quality control standards and in accordance with the Declaration of Helsinki.

### Ethical approval

Ethical approval was given by the Chinese Ethics Committee for Registering Clinical Trials (ChiECRCT) with the following reference number ChiECRCT-20090009.

### Ethics committee

Before the beginning of the study, all hospitals provided related data about the qualifications of the main researcher and the laboratory conditions in order to assist the ethics committee with their decision to allow the trial. The individual units were not required to request ethics approval again unless for a special request, and they were able to contact the ethics committee and state their request if necessary.

### Informed consent form

The informed consent form (ICF) was reviewed and approved by the ethics committees before the study commenced. Researchers informed participants of the relevant information in words and writing in clearly understandable language. The ICF was signed and dated by the participants or their representatives. Before signing the ICF, the participants and their representatives were allowed adequate time to read the form. The signed ICFs were maintained independently by the researchers and participants.

### Screening of the participants

The 400 prediabetes patients who were included according to the diagnosis criteria and inclusion criteria (see the next section) were divided into the treatment group and control group (1:1). All the participants signed the informed consent form.

### Intervention

For lifestyle intervention, the study team developed an individual diet program for each participant to ensure a reasonable proportion of carbohydrates, protein, and calories. In addition, the recommended diet was low in sugar and low in salt, and the program advised the participants to give up some bad habits. Smoking and alcohol consumption were strictly controlled. An exercise program was formulated for the participants according to age and sex.

The drug intervention covered a period of 12 months. All participants had to visit the researchers each month. The JQJT tablets for the treatment group required that the participants take seven tablets twice daily, and all drugs were taken before meals. The control group received a placebo, and the method and time were the same as for the treatment group.

### The model

The diagnosis criteria (The Diagnosis and Classification of Diabetes Mellitus, ADA 2008) [8] included impaired fasting glucose (IFG) (FPG 5.6 to 6.9 mmol/L) and/or impaired glucose tolerance (IGT)(FPG < 5.6 mmol/L and OGTT (oral glucose tolerance test) 2hPG (2-hour plasma glucose) 7.8 to 11.1 mmol/L).

To meet the inclusion criteria, the potential participant must have fulfilled the prediabetes diagnostic criteria, be 30 to 70 years of age, and have completed and submitted the informed consent form.

Exclusion criteria included a the history of diabetes (except gestational diabetes); recent cardiovascular events (severe organic heart disease, specific angina, type II degree atrioventricular block, etcetera); impaired hepatic and renal function; endocrine disease (cancer, hyperthyroidism, etcetera); former use of glucocorticoid, thiazide diuretics or nicotinic acid; pregnancy, preparation for pregnancy or currently lactating; suffering from mental diseases or non-cooperative as a patient; participated in other clinical trial within the last two weeks; or refusing to provide consent for the study.

Rejection criteria included failure to take medicine according to the protocol or incomplete data.

Suspension criteria included poor compliance; serious adverse events; complications and special physiological changes; abnormal unblinding; reluctance to continue the study; taking medicine forbidden in the study; or if national laws, the Ministry of Science and Technology, or other authorities decided to terminate the study, etcetera.

### Follow-up

The intervention period was 12 months, with participants checking in once a month for 12 months; after the intervention, the 12-month follow-up began and included check-ins in the 2nd, 6th, and 12th month after intervention.

### Outcome measures

The primary outcome was the occurrence of type 2 diabetes (according to the guidelines of the ADA, 2008) and blood glucose change to normal (FPG < 5.6 mmol/L and OGTT 2hPG < 7.8 mmol/L).

The secondary outcome was the score of the Short Form 36 Health Survey Questionnaire (SF −36).

Compliance was measured in participants according to the following procedure: researchers gave the investigational drugs to inpatients and explained the methods for using the medication to the outpatient. The compliance of the participants was determined by inspection of the remaining drugs and notes on the number remaining. The participants were asked to bring the drugs to the final visit to allow calculation of the number of tablets used.

The SF-36 was used as a measure of compliance; during the drug intervention, participants were required to complete five SF-36 forms, and in the follow-up, they were required to complete three forms. According to the clinical effects of the individual treatment, the actual number of forms completed differed.

Measures for the costs included the drug costs and lifestyle intervention costs. Lifestyle intervention costs consisted of lifestyle education materials costs (2.5 yuan per person for 24 months, 2 yuan per person in the intervention period), lifestyle education meeting costs (1220 yuan every meeting, with 80 meetings held during the drug intervention period and 150 meetings being held during the entire trial), and the costs of participants compliance (30 minutes to fill in the SF-36 form and 2 hours for listening to meetings. The current average monthly income of urban residents is 2400 yuan or 10 yuan per hour, which was used to calculate the cost of time).

The safety index includes a general medical examination; blood and urine tests during the study visits; functional examinations to determine EKG, ALT, BUN, and Cr; and adverse events.

### Model input data

The evaluation criteria of the efficacy of the treatment on the diabetes conversion rate was based on The Diagnosis and Classification of Diabetes Mellitus, ADA 2008.

$$ \mathrm{Incidence}\mathrm{of}\mathrm{diabetes}\mathrm{mellitus}=\mathrm{cases}\mathrm{of}\mathrm{diabetes}\mathrm{mellitus}\div total\mathrm{cases}\mathrm{of}\mathrm{each}\mathrm{group}\times 100\% $$

The rate of blood glucose return to normal (refer to the Diagnosis and Classification of Diabetes Mellitus, ADA 2008)

$$ \mathrm{Reversion}\mathrm{rate}=\mathrm{cases}\mathrm{o}\mathrm{f}\mathrm{blood}\mathrm{glucose}\mathrm{return}\mathrm{t}\mathrm{o}\mathrm{normal}\div \mathrm{total}\mathrm{cases}\mathrm{o}\mathrm{f}\mathrm{each}\mathrm{group}\times 100\% $$

### Compliance of participants

$$ \mathrm{Compliance}=\left[\left(\mathrm{dispensed}\hbox{-} \mathrm{remaining}\right)\div \mathrm{dispensed}\right]\times 100\% $$

#### The score of the SF-36

The SF-36 questionnaire was used to evaluate the various dimensions and entries, and between-group and within-group comparisons were made of the total score of the questionnaire. The score was calculated with measurement data.

### Costs

$$ \mathrm{The}\mathrm{drug}\mathrm{costs}=\left(\mathrm{dispensed}\hbox{-} \mathrm{remaining}\right)\times \mathrm{price}\mathrm{per}\mathrm{tablet}\times \mathrm{compliance} $$

The lifestyle intervention costs

$$ \begin{array}{l}=\left(\mathrm{lifestyle}\mathrm{education}\mathrm{materials}\mathrm{costs}+\mathrm{lifestyle}\mathrm{education}\mathrm{meetings}\mathrm{costs}+\mathrm{the}\mathrm{costs}\mathrm{of}\mathrm{participants}\mathrm{compliance}\right)\\ {}\times \mathrm{compliance}\mathrm{of}\mathrm{filling}\mathrm{in}\mathrm{the}\mathrm{S}\mathrm{F}\hbox{-} 36\times \mathrm{the}\mathrm{score}\mathrm{of}\mathrm{S}\mathrm{F}\hbox{-} 36\end{array} $$

#### The cost-effectiveness ratio

The incidence of diabetes mellitus was used to represent the clinical effectiveness index:

$$ \mathrm{the}\mathrm{cost}\hbox{-} \mathrm{effectiveness}\mathrm{ratio}=\mathrm{the}\mathrm{total}\mathrm{cost}\mathrm{s}\div \mathrm{incidence}\mathrm{of}\mathrm{diabetes}\mathrm{mellitus} $$

$$ \mathrm{the}\mathrm{cost}\hbox{-} \mathrm{effectiveness}\mathrm{ratio}=\mathrm{the}\mathrm{total}\mathrm{cost}\mathrm{s}\div \mathrm{reversion}\mathrm{rate} $$

### Statistical methods

For between-group comparisons, the following statistical tests were used: the chi-square test for enumeration data, the Wilcoxon rank sum test for non-normal distribution of measurement data, and the t-test for normal distribution of measurement data.

For dropping analysis, the chi-square test or Fisher exact probability method was used.

For compliance analysis, the chi-square test or Fisher exact probability method was used.

### Clinical outcomes and cost-effectiveness analysis

This clinical trial screened 400 patients, and 376 cases were qualified for inclusion. Ultimately, 362 cases were randomly assigned, with 182 cases in the treatment group and 180 cases in the control group. Adverse events in the treatment group and the control group occurred in five cases and three cases, respectively, so the incidences of adverse events were 2.78 % and 1.65 %. No statistically significant difference was observed between the two groups because there were no drug-related adverse reactions.

### Clinical outcomes

As shown in Tables 1, 2 and 3, the treatment group and control group showed no statistically significant difference (*P* > 0.05) at baseline for sex, marital status, nationality, height, weight, profession, history of disease, past medical history, blood sugar levels, values of OGTT, and so on. Only the baseline data for age showed slight differences (*P* = 0.036).The baseline data between the two groups were fundamentally equivalent and thus comparable at baseline.

**Table 1:** Comparison between the treatment group and control group in the baseline information

Baseline information	Index	The total cases	The control group	The treatment group
Age	N	362(0)	180(0)	182(0)
	Mean	54.49(8.78)	53.49(8.85)	55.49(8.61)
	Statistical	Wilcoxon rank sum test:-2.096		
	P	0.036		
Gender	Male n (%)	172(47.51 %)	82(45.56 %)	90(49.45 %)
	Female n (%)	190(52.49 %)	98(54.44 %)	92(50.55 %)
	N	362(0)	180(0)	182(0)
	Statistical	the Chi-square test:0.551		
	P	0.458		
Marital status	Unmarried n (%)	4(1.10 %)	2(1.11 %)	2(1.10 %)
	Married n (%)	358(98.90 %)	178(98.89 %)	180(98.90 %)
	N	362(0)	180(0)	182(0)
	Statistical	Fisher exact probability method		
	P	1.000		
Height	N	362(0)	180(0)	182(0)
	Mean	165.27(7.85)	165.17(7.65)	165.35(8.07)
	Statistical	Wilcoxon rank sum test:-0.205		
	P	0.837		
Weight	N	362(0)	180(0)	182(0)
	Mean	67.78(11.64)	67.49(11.21)	68.06(12.08)
	Statistical	Wilcoxon rank sum test:-0.062		
	P	0.837		
Profession	Worker n (%)	100(27.62 %)	49(27.22 %)	51(28.02 %)
	Farmer n (%)	29(8.01 %)	14(7.78 %)	15(8.24 %)
	Intellectual n (%)	100(17.62 %)	59(32.78 %)	41(22.53 %)
	Students n (%)	0(0.00 %)	0(0.00 %)	0(0.00 %)
	Retirement n (%)	94(25.97 %)	41(22.78 %)	53(29.12 %)
	Unoccupied n (%)	11(3.04 %)	4(2.22 %)	7(3.85 %)
	Other n (%)	28(7.73 %)	13(7.22 %)	15(8.24 %)
	N	362(0)	180(0)	182(0)
	Statistical	the Chi-square test:5.797		
	P	0.327

**Table 2:** Comparison of the history of disease and the past medical history between the two groups

Information	Index	The total cases	The control group	The treatment group
History of disease	No n (%)	269(74.31 %)	132(73.33 %)	137(75.27 %)
	Yes n (%)	93(25.69 %)	48(26.67 %)	45(24.73 %)
	N	362(0)	180(0)	182(0)
	Statistical	the Chi-square test: 0.179		
	P	0.673		
Past medical history	No n (%)	288(79.78 %)	145(80.56 %)	143(79.01 %)
	Yes n (%)	73(20.22 %)	35(19.44 %)	38(20.99 %)
	N	362(0)	180(0)	182(0)
	Statistical	the Chi-square test: 0.134		
	P	0.714

**Table 3:** Comparison between the two groups on fasting plasma glucose (FPG) and oral glucose tolerance test (OGTT) 2-hour plasma glucose (2hPG) in the screening stage

Information	Index	The total cases	The control group	The treatment group
FPG	N	362(0)	182(0)	180(0)
	Mean	6.03(0.60)	6.04(0.59)	6.03(0.60)
	Statistical	Wilcoxon rank sum test: 0.182		
	P	0.856		
OGTT 2hPG	Mean	8.86(1.34)	8.90(1.38)	8.82(1.30)
	Statistical	Wilcoxon rank sum test: 0.900		
	P	0.368		

Tables 4,5 and 6 showed the incidence of diabetes mellitus and the reversion rate. During the whole trial process, 37 cases of the treatment group advanced to diabetes, 59 cases of the control group advanced to diabetes (*P* < 0.05), therefore, the JinQi Jiangtang tablet effectively reduced the incidence of diabetes mellitus. In addition, 82 cases of the treatment group returned to normal blood glucose, and 54 cases of the control group returned to normal blood glucose (*P* < 0.05); therefore, compared with the control group, the JinQi Jiangtang tablet effectively enhanced reversion rate.

**Table 4:** The distribution of the numbers of cases in the 12-month intervention period

Group	Month 3			Month 6			Month 9			Month 12		
	NBG	DM	FO	NBG	DM	FO	NBG	DM	FO	NBG	DM	FO
The treatment group	37	8	9	20	5	2	7	6	0	12	11	1
The control group	25	23	9	18	13	5	1	5	2	6	11	0

*NBG* normal blood glucose cases, *DM* diabetes mellitus cases, *FO* fall off cases

**Table 5:** The incidence of diabetes mellitus and reversion rate in the 12-month intervention period

Group	The cases of diabetes	The incidence of diabetes mellitus (%)	The cases of normal blood glucose	Reversion rate (%)
The treatment group	30	16.5 %	76	41.8 %
The control group	52	28.9 %	50	27.8 %

Tables 7 and 8 showed the lifestyle intervention compliance. The two groups (treatment and control) separately received 681 SF-36 forms and 694 SF-36 forms, so the actual recovery rates of the SF-36 forms were 91.04 % and 83 %, respectively, in the intervention period of 12 months. In the 24-month follow-up period, the two groups separately received 1073 forms and 1077 forms, so the actual recovery rates of the SF-36 forms were 96.46 % and 87.37 %, respectively.

**Table 6:** The incidence of diabetes mellitus and the reversion rate in the 24-month period

Group	Cases of diabetes	Incidence of diabetes mellitus (%)	Cases of normal blood glucose	Reversion rate (%)
The treatment group	37	20.3 %	82	45.1 %
The control group	59	32.8 %	54	30 %

**Table 7:** The lifestyle intervention compliance in the 12-month intervention period

Group	Distribution copies	Recovery copies	The practical recovery of lifestyle intervention table (%)
The treatment group	681	620	91.04
The control group	694	576	83.00

Tables 9 and 10 showed the scores of SF-36. Despite the intervention period of 12 months or the whole period of 24 months, the average score of the two groups were more than 65 points, *P* > 0.05, so they had no significant difference.

**Table 8:** The lifestyle intervention compliance in the whole period of 24 months

Group	Distribution copies	Recovery copies	The practical recovery of lifestyle intervention table (%)
The treatment group	1073	1035	94.46
The control group	1077	941	87.3

**Table 9:** The scores of SF-36 in the 12-month intervention period

Group	$\overline{x}\pm S$	*t*	*P*
The treatment group	66.87 ± 6.45	−0.781	0.435
The control group	66.55 ± 6.08		

Table 11 showed the level of compliance of the participants. The compliance rates of the two groups were more than 80 % (*P* > 0.05) by rank sum test, so they had good compliance and no significant difference.

**Table 10:** The scores of SF-36 in the 24-month period

Group	$\overline{x}\pm S$	*t*	*P*
The treatment group	67.72 ± 6.06	−0.397	0.691
The control group	67.47 ± 5.53		

### Cost-effectiveness analysis

The costs were calculated according to the following:

1. For the drug costs, when one case advanced to diabetes mellitus or the blood glucose returned to normal, the case should have stopped taking medicine. The market retail price of the JQJT tablets (24 tablets × 3) was based on each tablet costing 0.26 yuan. The number of cases (participants) taking medicine at 3, 6, 9 and 12 months were 54, 27, 13 and 88 cases. Therefore, costs were determined as shown in the following equation:
  $$ \begin{array}{l}\mathrm{The}\ \mathrm{drugs}\ \mathrm{costs}=\left(7\times 3\times 30\times 3\times 54+7\times 3\times 30\times 6\times 27+7\times 3\times 30\times 9\times 13+7\times 3\times 30\times 12\times 88\right)\\ {}\times 0.26\times 92.94\%=227896.9\mathrm{yuan}\end{array} $$
2. The lifestyle intervention costs in the 12-month intervention period were calculated as indicated below:
  $$ \begin{array}{l}\mathrm{The}\ \mathrm{lifestyle}\ \mathrm{intervention}\ \mathrm{costs}\ \mathrm{of}\ \mathrm{the}\ \mathrm{treatment}\ \mathrm{group}=\left(182\times 2+1220\times 80/2+681\times 5+182\times 2\times 10 \times 80\right)\\ {}\times 91.04\%\times 66.87=20928123.4\mathrm{yuan}\end{array} $$
  $$ \begin{array}{l}\mathrm{The}\ \mathrm{lifestyle}\ \mathrm{intervention}\ \mathrm{costs}\ \mathrm{of}\ \mathrm{the}\ \mathrm{control}\ \mathrm{group}=\left(180\times 2+1220\times 80/2+694\times 5+180\times 2\times 10 \times 80\right)\\ {}\times 83\%\times 66.55=18815209.0\mathrm{yuan}\end{array} $$
3. The lifestyle intervention costs in the 24-month follow-up period were calculated as follows:
  $$ \begin{array}{l}\mathrm{The}\ \mathrm{lifestyle}\ \mathrm{intervention}\ \mathrm{costs}\ \mathrm{of}\ \mathrm{the}\ \mathrm{treatment}\ \mathrm{group}=\left(182\times 2.5+1220\times 150/2\times 1035\times 5+182\times 2 \times 10\times 150\right)\\ {}\times 94.46\%\times 67.72=41139933.9\mathrm{yuan}\end{array} $$
  $$ \begin{array}{l}\mathrm{The}\ \mathrm{lifestyle}\ \mathrm{intervention}\ \mathrm{costs}\ \mathrm{of}\ \mathrm{the}\ \mathrm{control}\ \mathrm{group}=\left(180\times 2.5+1220\times 150/2+941\times 5+180\times 2 \times 10\times 150\right)\\ {}\times 87.37\%\times 67.47=37529823.2\mathrm{yuan}\end{array} $$

The cost-effectiveness ratio is addressed in Tables 12 and 13, which showed the cost-effectiveness analysis for the 12-month intervention period. The total costs of the treatment group and the control group were 21,156,020.3 yuan and 1,885,209.0 yuan, respectively, and in terms of the reversion rate, the cost-effectiveness ratios were 506,124.9:676,806.1, so the treatment group was smaller. From the incremental cost-effectiveness ratio, the control group needed more than 167,213.7 yuan for each 1 % increase in the reversion rate to show that the treatment group had better economic advantages. With regard to the incidence of diabetes, the cost-effectiveness ratios of the treatment group and the control group were 1,282,183.0:651045.3, so the control group was smaller. From the incremental cost-effectiveness ratio, the treatment group needed more than 188,775.1 yuan for each 1 % decrease in the incidence of diabetes, which showed that the control group had the better economic advantage.

**Table 11:** Compliance of the participants

Group	$\overline{x}\pm S$	*Z*	*P*
The treatment group	92.94 ± 7.28	−0.771	0.441
The control group	92.27 ± 8.29

**Table 12:** The cost-effectiveness analysis in the 12-month intervention period (reversion rate)

Group	C (yuan)	E (%)	C/E	ΔC/ΔE
The treatment group	21156020.3	41.8	506124.9	
The control group	18815209.0	27.8	676806.1	167213.7

Tables 14 and 15 showed the cost-effectiveness analysis in the 24-month period. The total costs of the treatment group and the control group were 41,367,830.8 yuan and 37,529,823.2 yuan, and with regard to the reversion rate, the cost-effectiveness ratios were 917,246.8 and 1,250,994.1, respectively, so the treatment group was smaller. From the incremental cost-effectiveness ratio, the control group needed more than 254,172.7 yuan for each 1 % increase in the reversion rate to show that the treatment group had a better economic advantage. With regard to the incidence of diabetes, the cost-effectiveness ratios of the treatment group and the control group were 2,037,824.8 and 1,144,201.0, so the control group was smaller. From the incremental cost-effectiveness ratios, the treatment group needed more than 307,040.6 yuan for each 1 % decrease in the incidence of diabetes, which showed that the control group had a better economic advantage.

**Table 13:** The cost-effectiveness analysis in the 12-month intervention period (the incidence of diabetes)

Group	C (yuan)	E (%)	C/E	ΔC/ΔE
The treatment group	21156020.3	16.5	1282183.0	−188775.1
The control group	18815209.0	28.9	651045.3

**Table 14:** The cost-effectiveness analysis in the 24-month period (reversion rate)

Group	C(yuan)	E (%)	C/E	ΔC/ΔE
The treatment group	41367830.8	45.1	917246.8	
The control group	37529823.2	30	1250994.1	254172.7

### Sensitivity analysis

Cost-effectiveness results were most sensitive to the drug prices and resident incomes. Assuming that prices and resident incomes rose 5 %, the sensitivity analysis shows that the two group affected by the parameters changed little (Tables 16, 17, 18, and 19).

**Table 15:** The cost-effectiveness analysis in the 24-month period (the incidence of normal blood glucose)

Group	C (yuan)	E (%)	C/E	ΔC/ΔE
The treatment group	41367830.8	20.3	2037824.8	−307040.6
The control group	37529823.2	32.8	1144201.0	

*C* the total costs, *C/E* the cost-effectiveness ratio, *ΔC/ΔE* the incremental cost-effectiveness ratio

**Table 16:** The cost-effectiveness analysis in the 12-month intervention period (the reversion rate)

Group	C (yuan)	E (%)	C/E	ΔC/ΔE
The treatment group	22967057.6	41.8	549451.1	
The control group	20425193.3	27.8	734719.2	181561.7

**Table 17:** The cost-effectiveness analysis in the 12-month intervention period (the incidence of diabetes)

Group	C (yuan)	E (%)	C/E	ΔC/ΔE
The treatment group	22967057.6	16.5	1391942.9	−204989.1
The control group	20425193.3	28.9	706754.1

**Table 18:** The cost-effectiveness analysis in the 24-month period (the reversion rate)

Group	C (yuan)	E (%)	C/E	ΔC/ΔE
The treatment group	44911142.2	41.1	995812.5	
The control group	40740835.2	30	1358027.8	276179.3

**Table 19:** The cost-effectiveness analysis in the 24-month period (the incidence of diabetes)

Group	C (yuan)	E (%)	C/E	ΔC/ΔE
The treatment group	44911142.2	20.3	2212371.5	−333624.6
The control group	40740835.2	32.8	1242098.6	

## Results

The outcomes of the study contained two sections, namely, clinical outcomes and cost-effectiveness analysis outcomes.

The clinical outcomes revealed that the treatment group and control group showed no significant statistical differences (*P* > 0.05) in baseline values. The JinQi Jiangtang tablet effectively reduced the incidence of diabetes mellitus and enhanced the reversion rate. Compared with the control group (*P* < 0.05), the scores of the SF-36 of the two groups showed no significant difference (*P* > 0.05), and the compliance of participants showed no significant difference between the two groups.

With regard to the cost-effectiveness analysis outcomes, in the 12-month intervention period, for reversion rate, the treatment group had a better economic advantage according to the cost-effectiveness ratio and the incremental cost-effectiveness ratio. Regarding the incidence of diabetes, the control group had a better economic advantage according to the cost-effectiveness ratio and the incremental cost-effectiveness ratio. In the 24-month period, with regard to the reversion rate, the treatment group had a better economic advantage according to the cost-effectiveness ratio and the incremental cost-effectiveness ratio, and for the incidence of diabetes, the control group had a better economic advantage according to the cost-effectiveness ratio and the incremental cost-effectiveness ratio. Furthermore, these outcomes remained the same when assessed with sensitivity analysis. Assuming that prices and resident incomes rose 5 %, the sensitivity analysis shows that the two groups affected by the parameters changed little.

## Discussion

JinQi Jiangtang tablets have been confirmed as being a safe and effective modern drug for use in Traditional Chinese Medicine by clinical research in China and France, which was based on traditional prescription and optimized the modern pharmacology experimental methods. The JQJT tablets, composed by Coptis, Astragalusand Honeysuckle, show comprehensive treatment effects, such as decreasing blood pressure, serum lipids, and blood glucose; improving glucose tolerance; and when compared with a single antihypertensive, decreasing blood pressure. DPP, DREAM, and other large international clinical trials have confirmed that lifestyle intervention plays a very important role in the prevention of diabetes. Therefore, combining lifestyle intervention and Traditional Chinese Medicine intervention can achieve better effects, and this combination is easily accepted by patients. A foundation of good clinical screening; the use of prescriptions generally accepted as effective; and carrying out randomized, double-blinded, multicenter clinical research with large sample size are necessary. In addition, an evaluation of clinical medicine should consider three aspects, namely, safety, effectiveness, and economy. At the same time, the use of pharmacoeconomic evaluation started late in China, and the available TCM literature is insufficient; therefore, an economic evaluation standard is lacking. Obviously, in order to reveal the security, effectiveness and economy of the JQJT tablets combined with lifestyle intervention on the prevention and treatment of diabetes and to provide a basis for health decisions, it is necessary to explore a feasible evaluation method.

An evaluation of the cost-effectiveness of JinQi Jiangtang tablets for the prevention and treatment of diabetes was conducted using pharmacoeconomics combined with a parallel clinical trial of economics research. This study evaluated the efficiency and security of TCM, as well as the pharmaeconomics. This clinical trial screened 400 patients and identified 376 qualified cases. Ultimately, 362 cases were randomly assigned, with the treatment group receiving 182 cases and the control receiving 180 cases. The treatment group and the control group both received lifestyle education intervention, and then separately received JQJT tablets (treatment group) or JQIT simulation tablets (control). The two groups showed no significant statistical difference at baseline for sex, marital status, nationality, height, weight, profession, history of disease, past medical history, blood sugar levels, values of OGTT, and so on. The baseline data between the two groups were fundamentally equivalent and had baseline comparability. Adverse events occurred separately in the treatment group and the control group in five cases and three cases, respectively, so the incidences of adverse events were 2.78 % and 1.65 %. No statistically significant difference was observed between the two groups because there were no drug-related adverse reactions. At the same time, for the participants of the two groups, the study analyzed the clinical curative effect and the pharmacoeconomic aspects.

The use of cost-effectiveness analysis and incremental cost-effectiveness analysis revealed that the main cost of the treatment group included the drug cost and lifestyle education cost, and for the control group, the lifestyle education cost. With regard to the clinical outcomes, the incidence of diabetes mellitus and reversion rate were included. In the 12-month intervention period, the total costs of the two groups (treatment and control) were 21,156,020.3 yuan and 1,885,209.0 yuan, whereas the reversion rate and the incidence of diabetes were separately 41.8 % and 27.8 % and 16.5 % and 28.9 %. Therefore, the cost-effectiveness ratios were 506,124.9:676,806.1 and 1,282,183.0:651,045.3. The incremental cost-effectiveness analysis for the control group needed more than 167,213.7 yuan for each 1 % increase, and for the treatment group, more than 188775.1 yuan for each 1 % decrease in the incidence of diabetes was needed. In the 24-month period,, the total costs were 481,367,830.8 yuan and 37,529,823.2 yuan, respectively, and the reversion rate and the incidence of diabetes were separately 45.1 %, 30 % and 20.3 %, 32.8 %. Therefore, the cost-effectiveness ratios were 917,246.8:1,250,994.1 and 2,037,824.8:1,144,201.0. For the incremental cost-effectiveness analysis, the control group needed more than 254,172.7 yuan for each 1 % increase in the reversion rate, and the treatment group needed more than 307,040.6 yuan for each 1 % decrease in the incidence of diabetes.

Accordingly, in both the intervention period and the follow-up, the JinQi Jiangtang tablets combined with health education had a greater cost advantage effect than the health education alone on the normal blood glucose, and the health education had a greater cost advantage effect than the JinQi Jiangtang tablets combined with health education on the incidence of diabetes.

## Conclusions

By using pharmacoeconomics in combination with a clinical trial that had adopted a parallel research design method, the importance and effectiveness of lifestyle intervention alone, or in combination with JQJT tablets, in the prevention of diabetes was demonstrated. Not only did this combination treatment prevent the prediabetes from turning into diabetes, but also showed that the intervention could promote normal blood glucose. A cost-effectiveness analysis and an incremental cost-effectiveness analysis showed that JQJT tablets combined with lifestyle intervention offered a better economic advantage than lifestyle intervention alone for achieving normal blood glucose. In addition, the security, the curative effect, and the economic advantages of the JQJT tablets in preventing prediabetes from turning into diabetes have been proven and provide strong support for the ideas and theories of Traditional Chinese Medicine.

The cost-effectiveness analysis, whether concerned with the 12-month intervention period or the 24-month follow-up period, indicated that the lifestyle intervention had a better economic advantage than the JQJT tablets combined with lifestyle intervention, which was also proven by sensitivity analysis, but needs further discussion. (2×7×3×30×12×1.02=15422.4) (15422.4×20=308448yuan) 

In conclusion, effective interventions for prediabetes offer economic advantages by preventing or delaying the occurrence or development of diabetes and reduces the costs associated with complications of diabetes. This study has proven the necessity and importance of establishing a model for the economic benefits of TCM. Demonstrations of the economic benefits of TCM can promote the process of internationalization of TCM.

This clinical trial had some shortcomings, such as the sample size, which should have been larger, and a follow-up period insufficient to clarify the long-term curative effect and benefits of the JQJT tablets. In addition, the available literature for TCM pharmacoeconomics evaluation is limited, and the standards for pharmacoeconomic evaluation are currently being debated in China. However, the cost-effectiveness analysis is valuable to pharmacoeconomics evaluations, even though the study may need further discussion. This research provides a valuable reference for pharmacoeconomic evaluation of TCM and makes a positive contribution to the standardization of an evidence-based pharmacoeconomic evaluation process for TCM.

## Additional files

Additional file 1:
**CONSORT 2010 Flow Diagram.** (DOC 46 kb) Additional file 2:
**CONSORT 2010 checklist.** (DOC 219 kb)

## Abbreviations

ALT: alanine transaminase
AST: aspartate aminotransferase
BUN: urea nitrogen
Cr: creatinine
ICF: informed consent form
JQJT: JinQi Jiang Tang
OGTT: oral glucose tolerance test
RCT: randomized controlled clinical
SF-36: Short Form 36 Health Survey Questionnaire
TCM: Traditional Chinese Medicine
