# Supplementary material for: The cost-effectiveness analysis of JinQi Jiangtang tablets for the treatment on prediabetes: a randomized, double-blind, placebo-controlled, multicenter design
Source: Trials. 2015 Nov 3;16:496. doi: 10.1186/s13063-015-0990-9 (PMC4632405; doi:10.1186/s13063-015-0990-9)
Supplement: Additional file 1: — CONSORT 2010 Flow Diagram. (DOC 46 kb) [file 13063_2015_990_MOESM1_ESM.doc]

**
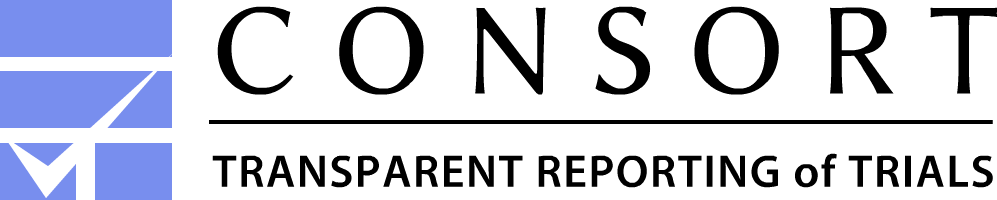
**

**CONSORT 2010 Flow Diagram**

**Allocation**

**Analysis**

**Follow-Up**

**Enrollment**

Assessed for eligibility (n=639 )

Excluded (n=267 )

♦  Not meeting inclusion criteria (n=253)

♦  Declined to participate (n=0 )

♦  Other reasons (n=14 )

Analysed (n=152 )
♦ Excluded from analysis (give reasons) (n=0 )

Lost to follow-up (give reasons such as on business or lose their message) (n=12 )

Discontinued intervention (give reasons like adverse event) (n=1 )

Allocated to intervention (n=182 )

♦ Received allocated intervention (n=165 )

♦ Did not receive allocated intervention (give reasons) (n=17 )

Lost to follow-up (give reasons such as on business or lose their message) (n=15 )

Discontinued intervention (give reasons adverse event) (n=3 )

Allocated to intervention (n=180 )

♦ Received allocated intervention (n=166 )

♦ Did not receive allocated intervention (give reasons) (n=14 )

Analysed (n=148 )
♦ Excluded from analysis (give reasons) (n=0 )

Randomized (n=362 )
